# High-frequency cyber-pornography exposure and gender as influencing factors in adult problematic pornography use following minor-onset viewing

**DOI:** 10.3389/fpsyt.2026.1815849

**Published:** 2026-04-21

**Authors:** Pantxika Victoire Morlat, Maria Limniou, Miya Dobbie, Stavros Chatzisymeonidis

**Affiliations:** Department of Psychology, University of Liverpool, Liverpool, United Kingdom

**Keywords:** cyber-pornography, early pornography exposure, gender, problematic pornography use, sexual orientation, sexual permissiveness

## Abstract

**Introduction:**

Due to the increased ease of access to cyber-pornography (C-P) and the decreasing age of consumers, investigations into this topic are fundamental to improve timely understanding of C-P and what its use entails. Research has yet to investigate the relationship between problematic pornography use (PPU), frequency of cyber-pornography exposure (FC-PE) and sexual permissiveness (SP) in adults with first exposure to C-P before the age of 18. This study investigated (i) the association between PPU and FC-PE and (ii) the association between PPU and SP while testing gender and sexual orientation as potential moderators.

**Materials and methods:**

The sample of this study consisted of 200 individuals (51.5% women, 48.5% men) aged 18-58 years old (*M_age_* = 21.67 years, *SD* = 5.22) who reported sustained C-P use with first exposure before the age of 18. Participants identified as heterosexual (61.0%), bisexual (26.5%), homosexual (6.0%) and ‘other sexual orientation’ (6.5%). Measures included the Problematic Pornography Consumption Scale, the Brief Sexual Attitudes Scale and demographic items.

**Results:**

Linear regression analyses indicated a very strong positive association between PPU and FC-PE (*p* < 0.001), while the association between PPU and SP was weak, negative and only trend-level (*p* = 0.067). Although neither moderation effect reached significance, gender emerged as a significant predictor of PPU (*p* < 0.001) with men reporting substantially higher PPU than women, whereas sexual orientation was a trend-level predictor of PPU (*p* = 0.064).

**Conclusion:**

The findings highlight the need for greater awareness and monitoring of C-P use, as high-frequent exposure may signal elevated risk for PPU, particularly among men. Future research is warranted to clarify the roles of SP and sexual orientation in PPU. Early identification and targeted interventions in clinical settings and educational programs may help mitigate negative outcomes, reduce stigma and promote healthier sexual attitudes and self-regulation, an increasingly important priority given the rising prevalence of C-P exposure among minors.

## Introduction

Recent regulatory changes in the United Kingdom (UK) highlight growing public health concerns surrounding minors’ access to cyber-pornography (C-P). Under the Online Safety Act, C-P websites will be required to implement strict age-verification systems from July 2025 (1). Simultaneously, the UK Government has introduced a ban on the depiction of strangulation in pornography, effective from November 2025, designated such content as a priority offence. Platforms will be obligated to take proactive measures to prevent users from accessing illegal strangulation and suffocation material (2). These regulations reflect increasing recognition that unrestricted Internet access, combined with insufficient safeguards for young users has intensified concerns about early and frequent C-P exposure.

C-P is defined as “professionally produced or user-generated (audio)visual material on or from the Internet that typically intends to arouse the viewer and depicts sexual activities and (aroused) genitals in unconcealed ways, usually with close-ups on oral, anal, and vaginal penetration” (3, p. 751). Its availability has expanded rapidly due to high−speed Internet access and the proliferation of large platforms such as Pornhub and XVideos, which receive millions of daily visits (4, 5). This is particularly concerning given that the average age of first exposure is estimated to be between 11 to 13 years old (6). A recent Ofcom (1) report found that 8% of UK minors aged 8 to 14 accessed C-P within a single month, including approximately 3% of children aged 8-9.

Ease of accessing C-P, including by young users, urges updated research on C-P and its associated risks. The degrading nature of certain scenes, sometimes depicting illegal behaviour, may transfer into normalisation of problematic sexual practices and gendered expectations of sexual attitudes and behaviours (7, 8). Thus, as illustrated by recent governmental actions into monitoring C-P and increased protection of users (1, 2), this research follows this movement by developing contemporary understanding of C-P and its associated patterns with an overreaching goal of promoting on- and off-screen wellbeing. This study is aimed at providing updated findings on C-P that may be useful for policies, clinical interventions and future research sharing a common goal of protecting users, especially young ones. Although prior research has explored associations between pornography use, gender, sexual orientation and sexual permissiveness (SP) in adulthood (9–11), there is lack of knowledge about whether these patterns persist among adults whose C-P use started before age 18. Accordingly, this study takes a first step, analysing a sample of 200 adults, to examine i) how the frequency of C-P exposure (FC-PE) relates to problematic pornography use (PPU) in individuals who first viewed C-P as minors and ii) whether gender and sexual orientation moderate the relationship between SP and PPU. As recently noted by (12), further research on gender differences among individuals exposed to C-P during adolescence is essential for developing targeted prevention strategies.

### Problematic pornography use and frequency of cyber-pornography exposure

PPU is characterised by difficulty regulating sexually explicit media use, which may involve escalating consumption, impaired impulse control or negative consequences, such as distress and interpersonal difficulties (13). Meta-analytic evidence suggests a pooled PPU prevalence of approximately 13.0% (14). PPU is often conceptualised as a subtype of hypersexual or compulsive sexual behaviour disorder (CSBD; 15–17) and has been linked to risky sexual behaviours and, in some cases, in-person sexual aggression (17, 18). Prior work indicates a positive association between PPU and FC-PE (19, 20), with early research suggesting that consuming more than 11 hours of pornography per week may indicate addictive patterns (21).

Concerns about C-P content extend beyond frequency of exposure, Fritz et al. (8) reported 45.0% of Pornhub scenes and 35.0% of XVideos scenes contained at least one act of physical aggression. Some videos labelled as “feminist”, despite the term’s typical association with gender equality, depict violence overwhelmingly perpetrated by men against women, representing a form of aggression directing at women who advocate for equal rights (22, 23). Reflecting these concerns, the Minister for Victims and Tackling Violence Against Women and Girls stated that the government “will not stand by whilst women are violated online and victimised by violent pornography which is allowed to normalise harm” (2). Common aggressive acts include spanking, gagging, slapping, hair pulling, choking, use of degrading language (8, 24) and condom use barely depicted (25). In extreme cases, prolonged C-P exposure may contribute to desensitisation, preference for more extreme content or engagement with illegal material such as indecent images of children (IIOCs; 26).

Given these concerns, the present study investigates the relationship between FC-PE and PPU among adults who were first exposed to C-P as minors and descriptively gathered participants’ preferred type of C-P content.

### Problematic pornography use and sexual permissiveness

Pornography commonly depicts sexual activity between uncommitted partners, emphasising casual encounters and self-gratification (11). This portrayal aligns with the construct of SP, defined as favourable attitudes toward multiple sex partners, casual sex or non-monogamy - attitudes that may shape behaviour in sexual/erotic situations (27). Concerns have been raised that C-P’s frequent depiction of permissive sex acts may contribute to increase SP among users, including minors (10). A recent systematic review reported associations between C-P exposure and earlier sexual debut (before age 16), greater numbers of sexual partners, group sex, sexual aggression and other risky sexual behaviours during adolescence (28). However, findings in this area remain mixed. Some studies have found no reliable longitudinal associations between pornography use and increase SP (10). While Lewczuk et al. (29) provide the only evidence that SP predicts PPU in both men and women, although their study did not clarify whether participants’ exposure began before adulthood.

The current study expands on the current literature by examining the relationship between SP and PPU among adults who first viewed C-P before the age of 18.

### Problematic pornography use, sexual permissiveness and gender

A “pornography gender gap” indicates that men are significantly more likely than women to consume and be exposed to C-P (30, p. 152). C-P is predominantly produced to elicit sexual arousal in men, often in the context of masturbation (31). Gender differences also emerge in self-reporting C-P use: women tend to underreport whereas men tend to overreport (32). Recent findings suggest that first exposure typically occurs at age 13 for boys and age 14 for girls (33), with boys aged 13-14 significantly more likely to access C-P than girls of the same age (1). Men also report seeking out and encountering more extreme C-P content whereas women’s exposure is more often accidental (34).

Qualitative research with men investigated for accessing IIOCs in the UK suggests that some individuals describe becoming ‘bored’ of general adult pornography and escalating to more extreme content, often due to easy access on adult pornography websites (35). Emphasising the risks for young people, half of these men (*n* = 10) reported first exposure to pornography at an early age. Men are also more likely to report PPU, with 11.0% identifying as addicted compared to 3.0% of women (36), who are more likely to report being in relationships with partners exhibiting PPU (37). These findings are consistent with the notion that C-P may both reflect and reinforce sexual norms, and that gender differences in consumption could be shaped by underlying sexual attitudes, particularly SP. Consistent with this perspective, (11) reported that pornography consumption was associated with higher SP, with men endorsing more permissive attitudes than women. Conversely, (10) identified that the association between pornography use and SP was significant only among adolescent girls.

Considering these gender-based differences in C-P consumption and SP, the present study examines gender as a potential moderator of the relationship between SP and PPU.

### Problematic pornography use, sexual permissiveness and sexual orientation

Patterns of C-P consumption also vary across sexual orientations. Specifically, gay and bisexual men report more frequent pornography use than heterosexual men (38), and sexual minority men and women tend to exhibit higher levels of PPU than their heterosexual peers (39). Sexual minority individuals also report high levels of self-perceived pornography addiction (40). Additionally, LGBTQ^1^ males scored higher on hypersexuality indicators, including frequency of pornography viewing, than LGBTQ females (9). (41) clarify these findings by concluding that bisexual and uncertain men reported higher hypersexuality than heterosexual men, which may reflect more permissive sexual attitudes. Sexual minority men also tend to report more permissive-alike sexual behaviours, including a greater number of sexual partners and more frequent casual sexual encounters, compared to sexual minority women and heterosexual individuals (9).

Given these established associations between sexual orientation, SP and PPU, the present study includes sexual orientation as a moderator of the relationship between PPU and SP.

### The current study

Although frequent C-P exposure has been linked to PPU (e.g., 19, 20), this association remains understudied among adults whose exposure began before age 18. While prior research has examined pornography use in relation to gender, sexual orientation and SP (9–11), few studies have connected these factors to PPU. To the authors’ knowledge, only Lewczuk et al. (29) found SP predicted PPU in both men and women and their study did not consider developmental onset of exposure.

The present study addresses this gap by examining, among adults who first accessed C-P before age 18, i) the association between FC-PE and PPU 18 and ii) the association between SP and PPU, determining whether this relationship is moderated by gender and sexual orientation.

Accordingly, four hypotheses were tested:

*H1*: FC-PE is positively associated with PPU.
*H2*: SP is positively associated with PPU.
*H3*: Gender moderates the relationship between SP and PPU.
*H4*: Sexual orientation moderates the relationship between SP and PPU.

## Methods

### Participants

Participants were recruited via volunteer sampling using social media advertisements (Instagram, Snapchat, Reddit and WhatsApp), campus posters and the University’s participation point scheme. None of the advertisements were paid for and participants did not receive financial compensation. Inclusion criteria were age ≥ 18 years, fluency in English and self-reported exposure to C-P before age 18. Information on participants’ country of residence was not collected. Exclusion criteria were incomplete surveys.

The final sample comprised 200 participants (51.5% women, 48.5% men) aged 18-58 years old (*M_age_* = 21.67 years, *SD* = 5.22). Regarding their sexual orientation, 61.0% of the participants identified as heterosexual, 26.5% as bisexual, 6.0% as homosexual and 6.5% as ‘other sexual orientation’.

Most of the participants were exposed to C-P several times a week (23.0%), followed by less than once a month (19.0%), once a week (15.0%), once a day (12.0%), once a month (11.0%). Once a fortnight and several times a day were equally represented (10.0%). Regarding content, the majority reported viewing non-violent and non-degrading content (58.5%) and 41.5% of the participants reported typically viewing violent (i.e., choking, slapping) or degrading content (i.e., name-calling). By gender, 54.37% of women and 62.89% of men reported non-violent content, and 45.63% of women and 37.12% of men reported violent content.

The final sample of 200 participants exceeded common recommendations for regression analyses. Based on an *a priori* power analysis conducted in G*Power (42), detecting a medium effect size (*f²* = 0.15) with *α* = 0.05 and 80% power requires approximately 55 participants for simple linear regression (one predictor) and 77 participants for multiple regression with three predictors. The present sample, therefore, provided adequate statistical power for all planned analyses, including moderation models examining gender and sexual orientation as moderators of the association between SP and PPU.

### Materials

Data were collected anonymously using the Qualtrics survey online platform (www.qualtrics.com). The average completion time was 15-20 minutes. The survey recorded demographic variables (age, gender and sexual orientation) and the following items and scales.

#### Frequency of cyber-pornography exposure

Four bespoke items assessed lifetime exposure, age at first exposure, typical viewing frequency and typical content type. Frequency was coded on an ordinal scale (1= Less than once a month to 7= Several times a day). Content type was coded dichotomously (non-violent/non-degrading versus violent/degrading).

#### Problematic pornography consumption scale (PPCS)

The PPCS (43) is an 18-item self-report scale used to measure participants’ levels of PPU. Items are rated on a 7-point Likert scale (1 = Never to 7 = All the time) and summed to produce a total score (range 18-126). A score ≥ 76 is commonly used as a threshold for possible PPU. The validity of PPCS has been established in various populations, including Chinese and Hungarian men (44) and Spanish-speaking adolescents (45), and its internal consistency was excellent for this sample (*α* = .96).

#### Brief Sexual Attitudes Scale – Permissiveness subscale (BSAS Permissiveness)

A total of 10 items from the BSAS (27) assessed SP. Participants were asked how much they agree with each statement, whilst keeping their current partner in mind or their most recent partner in mind or what they think their responses would most likely be. Answers range from 1 (Strongly disagree) to 5 (Strongly agree). The validity of BSAS has been demonstrated across diverse populations, such as Filipino men (46) and Turkish-speaking university students (47). The subscale demonstrated acceptable internal consistency based on participants’ responses (*α* = .66).

### Procedure

Initially, participants were required to read an electronic information sheet outlining the study’s purpose, inclusion criteria, data protection information and researchers’ contact details. Upon providing informed consent, participants answered three demographic questions followed by four questions related to their use of C-P. Subsequently, they completed the two standardised measures (PPCS and BSAS Permissiveness). Finally, all participants were fully debriefed, including contact details for the study and relevant support organisations.

### Design

A cross-sectional correlational design examined associations between i) FC-PE and PPU and ii) SP and PPU with gender and sexual orientation tested as moderators. The analyses comprised linear regressions to examine direct associations, and moderation analyses to explore potential interactive effects. The main predictors were examined in separate models to evaluate their independent association with PPU, providing a clearer interpretation of each predictor. In the study, PPU served as the dependent variable while FC-PE and SP were treated as independent variables. Gender and sexual orientation were included as moderators to assess whether the relationships between SP and PPU varied across these demographic characteristics. This design allowed for the simultaneous examination of both direct and conditional effects on PPU in adults with minor-onset exposure to C-P.

### Analysis

Data were analysed using RStudio (48) computer software. Linear regression analyses and moderation analyses were conducted to examine the four hypotheses. The significance level was fixed at *p* < 0.05 for all analyses. Preliminary diagnostics were incorporated to check outliers, missing data and normality of distribution. Less than 5% of cases had missing data, and no systematic patterns were observed. Missing data were excluded using listwise deletion. Data quality checks indicated that no automated or suspicious responses were identified in the sample.

## Results

All regression assumptions were examined and met, including normality of residuals, linearity, independence of errors, homoscedasticity and absence of multicollinearity.

### *H1*: problematic pornography use and frequency of cyber-pornography exposure

**Figure 1:**
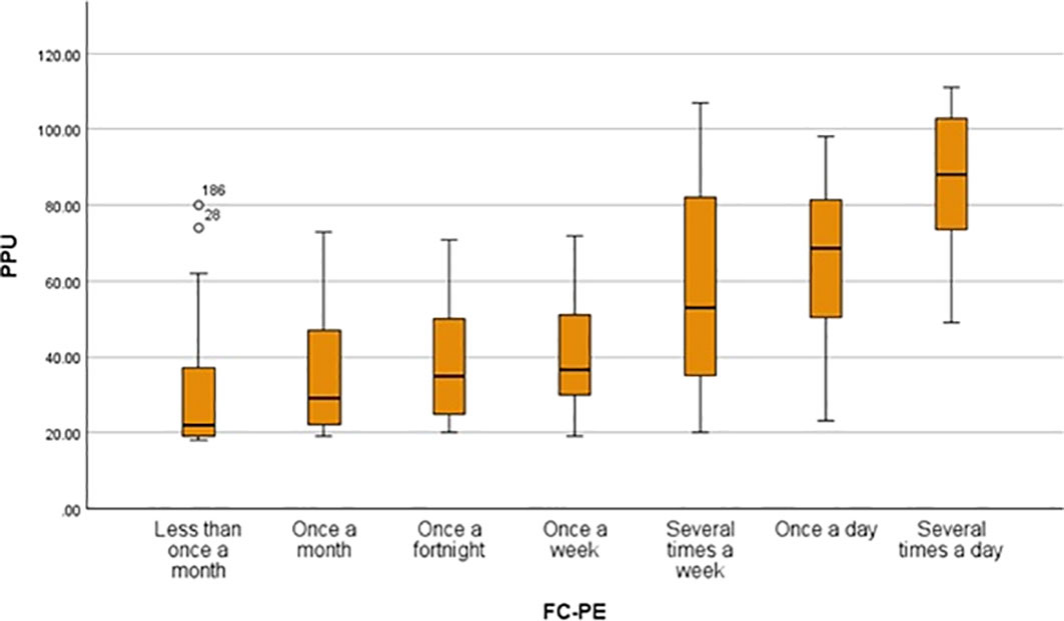
Problematic pornography use by frequency of cyber-pornography exposure. PPU, Problematic Pornography Use; FC-PE, Frequency of Cyber-Pornography Exposure.

A simple linear regression was conducted to examine whether FC-PE predicted PPU. The model was statistically significant and predicted approximately 37.9% of the variance in PPU, *adjusted R²* = 0.379, *F* (1, 198) = 122.4, *p* < 0.001. A positive association was found between PPU and FC-PE, *B* = 8.15, *β* = 0.62, *SE* = 0.74, *t* (198) = 11.06, *p* < 0.001, 95% CI [6.70, 9.61], with a very large effect size (*f²* = 0.61). This indicates that for every-one unit increase in FC-PE, PPU increased by 8.15 points. Descriptively, PPU scores rise consistently across exposure categories (e.g., Less than once a month: *M* = 30.18, *SD* = 16.34; Once a month: *M* = 36.18, *SD* = 17.23; Once a fortnight: *M* = 37.30, *SD* = 14.25; Once a week: *M* = 40.73, *SD* = 16.57; Several times a week: *M* = 56.41, *SD* = 26.11; Once a day: *M* = 65.63, *SD* = 21.25; Several times a day: *M* = 85.50, *SD* = 19.48). As shown in **Figure 1**, PPU increased with higher FC-PE. These results support *H1*.

### *H2:* problematic pornography use and sexual permissiveness

**Figure 2:**
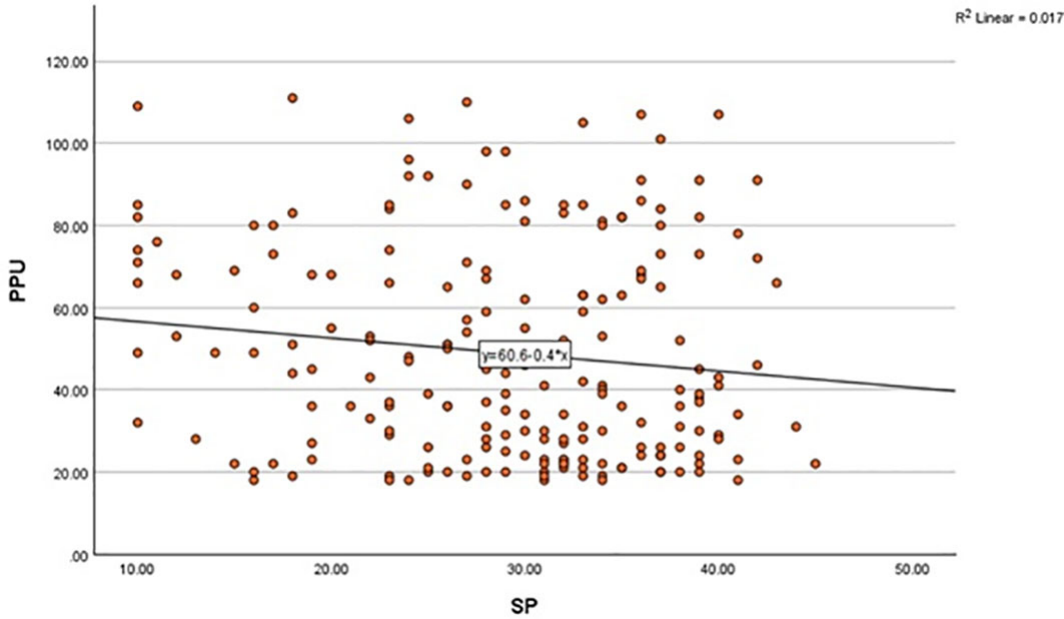
Trend-level negative association between problematic pornography use and sexual permissiveness. PPU, Problematic Pornography Use; SP, Sexual Permissiveness.

A simple linear regression was conducted to examine whether SP predicted PPU. The model was not statistically significant, *adjusted R²* = 0.012, *F* (1, 198) = 3.39, *p* = 0.067. A trend-level negative association was observed, *B* = -0.40, *β* = -0.13, *SE* = 0.22, *t* (198) = -1.84, *p* = 0.067, 95% CI [-0.83, 0.03], with a very small effect size (*f²* = 0.01). Because the association did not reach statistical significance, *H2* cannot be supported. **Figure 2** illustrates the trend-level negative association between PPU and SP.

### *H3*: gender as a moderator of the interaction between problematic pornography use and sexual permissiveness

**Figure 3:**
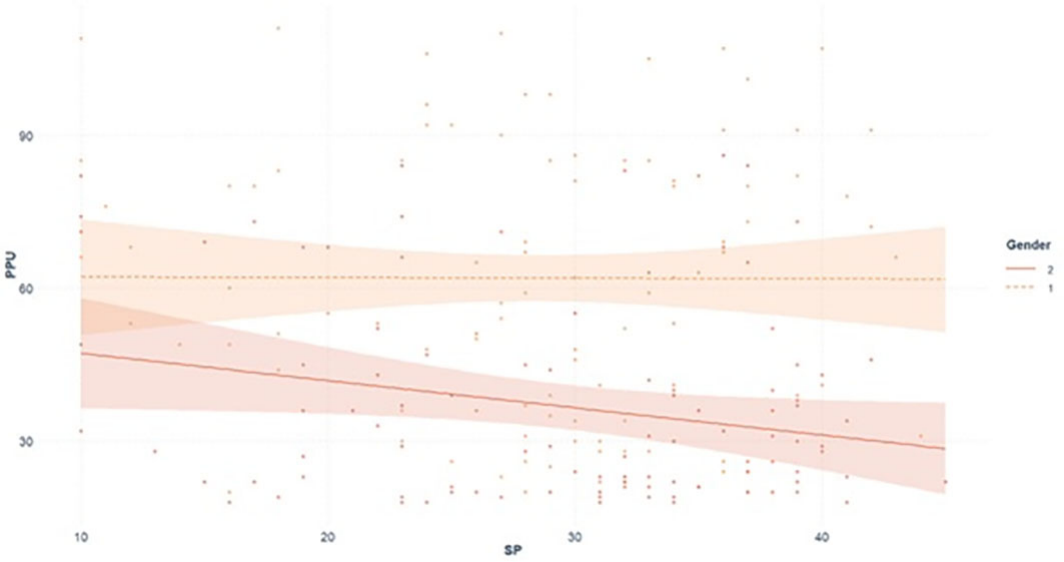
Predicted values of problematic pornography use by sexual permissiveness and gender. PPU, Problematic Pornography Use; SP, Sexual Permissiveness; 1 = Men; 2 = Women.

In step 1, SP and gender significantly predicted 23.8% of the variance, *adjusted R²* = 0.238, *F* (2, 197) = 32.11, *p* < 0.001. SP was not a significant predictor, *B* = -0.30, *β* = -0.10, *SE* = 0.19, *t* (197) = -1.57, *p* = 0.118, 95% CI [-0.68, 0.07], whereas gender showed a strong negative association with PPU, *B* = -24.83, *β* = -0.48, *SE* = 3.21, *t* (197) = -7.35, *p* < 0.001, 95% CI [-31.16, -18.50], reflecting a substantial difference in PPU scores between men (*M* = 61.92, *SD* = 25.76) and women (*M* = 36.75, *SD* = 19.43). The step 1 model indicated a medium effect size (*f²* = 0.33). In step 2, adding the SP x gender interaction explained an additional 0.7% of the variance, *ΔR²* = 0.007, *adjusted R²* = 0.241, *F* (3, 196) = 22.11, *p* < 0.001. However, the interaction term (SP x gender) was not significant, *B* = -0.52, *β* = -0.41, *SE* = 0.39, *t* (196) = -1.36, *p* = 0.177, 95% CI [-1.28, 0.24]. Therefore, gender did not moderate the association between SP and PPU, and *H3* was not supported. **Figure 3** displays the interaction between SP and gender predicting PPU, although the plotted slopes appear to differ, the interaction effect was not statistically significant.

### *H4*: sexual orientation as a moderator of the interaction between problematic pornography use and sexual permissiveness

**Figure 4:**
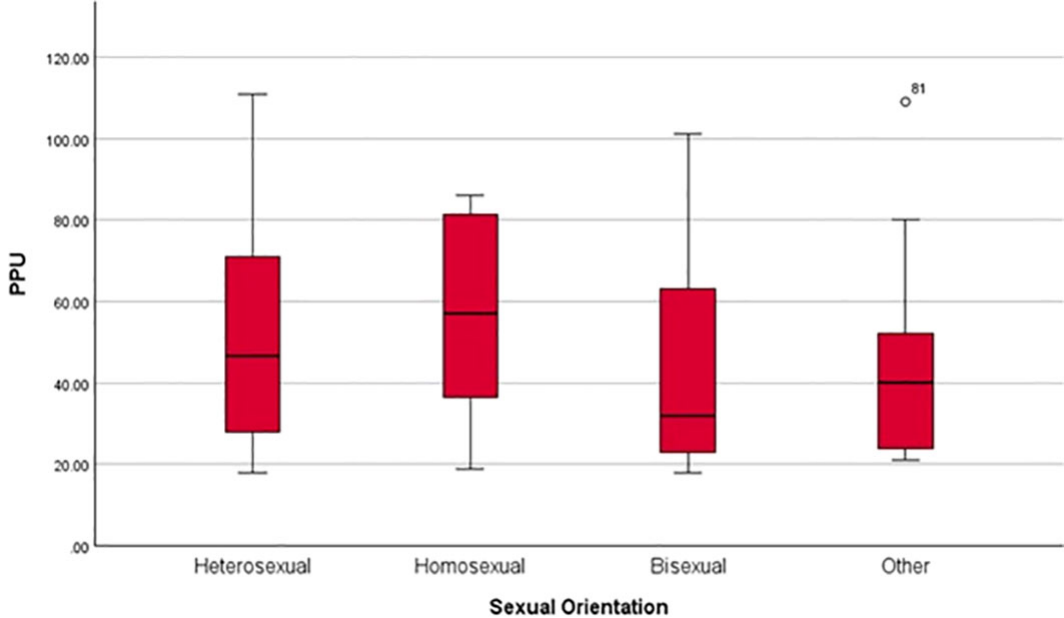
PPU, problematic pornography use by sexual orientation.

In step 1, SP and sexual orientation significantly predicted PPU, and explained 2.4% of variance, *adjusted R²* = 0.024, *F* (2, 197) = 3.45, *p* = 0.034. Neither SP (*B* = -0.39, *β* = -0.13, *SE* = 0.22, *t* (197) = -1.80, *p* = 0.074, 95% CI [-0.82, 0.04]) nor sexual orientation (*B* = -3.23, *β* = -0.13, *SE* = 1.74, *t* (197) = -1.86, *p* = 0.064, 95% CI [-6.66, 0.19]) were significant predictors of PPU, and the overall model reflected a small effect size (*f²* = 0.03). Sexual orientation was coded as: 1 = Heterosexual; 2 = Homosexual; 3 = Bisexual; 4 = Other sexual orientation. Mean PPU scores varied across sexual orientation groups (Heterosexual: *M* = 51.37, *SD* = 26.54; Homosexual: *M* = 56.08, *SD* = 26.01; Bisexual: *M* = 42.72, *SD* = 23.69; Other sexual orientation: *M* = 45.15, *SD* = 26.56). In step 2, adding SP x sexual orientation interaction explained an additional 0.1% of the variance, *ΔR²* = 0.001, *adjusted R²* = 0.020, *F* (3, 196) = 2.39, *p* = 0.070. The interaction term (SP x sexual orientation) was not significant, *B* = -0.10, *β* = -0.14, *SE* = 0.19, *t* (196) = -0.55, *p* = 0.582, 95% CI [-0.47, 0.27], indicating that sexual orientation did not moderate the association between SP and PPU. Therefore, *H4* was not supported. **Figure 4** illustrates PPU scores across sexual orientation groups. However, due to the imbalance in the number of participants across sexual orientation groups, the findings have limited generalisability and should be interpreted with caution. Participants from all sexual orientation groups were retained despite smaller subgroup sizes (e.g., *n* = 12 identifying as homosexual and *n* = 13 as ‘other sexual orientation’), as excluding these cases could reduce the representativeness of the sample and introduce potential bias. This approach is consistent with the emphasis on transparency supported by the American Psychological Association (49).

## Discussion

This study examined the relationships between PPU, FC-PE, SP, gender and sexual orientation in a sample of adults aged 18-58 who were exposed to C-P as minors. The first aim was to explore the association between PPU and the FC-PE. The second aim was to explore the association between SP and PPU and whether this relationship was moderated by gender or sexual orientation. Four hypotheses were tested using regression and moderation analyses, the findings are discussed subsequently. Overall, FC-PE and gender significantly predicted PPU, whereas SP and sexual orientation showed trend-level negative associations with PPU. Although these latter findings did not reach statistical significance, their proximity to the threshold warrants interpretive consideration.

The linear regression results supported a robust association between PPU and FC-PE, aligning with addictive behaviour theories (e.g., Positive Reinforcement Loop Theory, 50). These findings are consistent with earlier work suggesting that extensive pornography use (i.e., 11 hours per week) may indicate problematic or addictive patterns (21). In the present study, participants who viewed C-P several times a day had a mean score of PPU exceeding 76, whereas those with lower viewing frequencies scored below this threshold. This pattern reflects well-established neurobiological mechanisms. Indeed, repeated exposure to rewarding stimuli produces dopamine surges in the mesolimbic pathway, reinforcing the behaviour. Over time, ‘neuroadaptation’ reduces natural dopamine responsiveness, contributing to tolerance and dependence, escalating consumption (51, 52). Prior research similarly reports significant associations between PPU and FC-PE (20), suggesting that prolonged or frequent C-P consumption may evolve into problematic behavioural patterns (53). Therefore, the present findings highlight the importance of consumption duration and frequency in the development of PPU. As noted in previous literature, PPU may manifest through impaired impulse control, emotional distress and interpersonal difficulties, with negative consequences for both users and their close social networks (54). Beyond frequency, however, the nature of the content consumed may also shape the development of problematic use.

Drawing on Cultivation Theory (55), repeated exposure to C-P may influence individuals’ perceptions of normative sexual behaviour, potentially encouraging the replication of observed behaviours in real-life scenarios. C-P consumption has been linked to risky sexual behaviours and unrealistic expectations of oneself and sexual partners. According to _3_AM Theory (56), individuals may acquire new sexual scripts through media exposure (‘Acquisition’), which can later be triggered by similar cues (‘Activation’) and eventually enacted in real-life sexual situations (‘Application’). Given that 35.0-45.0% of content on major C-P platforms depicts violence (8), consumers may internalise such portrayals as typical or desirable aspects of sexuality. Notably, similar patterns were present in this sample, with 41.5% reporting that they primarily consumed violent or degrading C-P. Additional factors, such as gendered motivations, attraction to performers, situational influences (e.g., sexual attitudes) and ease of access may further facilitate the ‘Application’ of C-P scripts (57). The latter factor is particularly salient in this study, as all participants reported being exposed to C-P before reaching legal adulthood, highlighting the accessibility of such material to minors. In this context, legislative efforts such as the Online Safety Act, which aims to restrict minors’ access to C-P and violent content (1), may help reduce PPU and associated risky sexual behaviours by disrupting the sequential processes of ‘Acquisition’, ‘Activation’ and ‘Application’. Nevertheless, sexual attitudes may also play a role in the development of PPU, motivating the examination of the relationship between PPU and SP in this study.

While prior research has examined the relationship between sexual attitudes and C-P consumption, the specific link between PPU and SP has received comparatively less attention (e.g., 29). In that study, SP predicted PPU in both men and women, whereas the present findings revealed only a trend-level negative association. This lack of significance may reflect limited statistical power, but it also highlights the need for further research on how gender may shape the relationship between SP (and potentially other sexual attitudes) and PPU. However, it is important to consider developmental onset of exposure, especially in relation to the increased digital literacy of young people (58). Broader shifts in sexual and behaviours, promoting ‘hook-up’ culture as often normalised and expected by young people (59), may lead to more permissive sexual attitudes and impact the identification of maladaptive functioning related to C-P. Ybarra et al. (60) claim that for youth aged 10-15 years old: “intentional exposure to violent x-rated material over time predicted an almost 6-fold increase in the odds of self-reported sexually aggressive behavior” (p. 1). The growing reliance on digital platforms positioned the Internet as an expected source for sexual development and knowledge and overall sex education (61, 62). Understanding of PPU may therefore be scarce, as it may not contradict believed normalised sexual behaviours, including C-P viewing, especially if it occurs from a young age. Individuals’ internalised beliefs may influence their C-P consumption patterns without necessarily escalating to problematic use. Moreover, early exposure and long-term engagement may normalise C-P consumption, reducing the extent to which SP influences the development of PPU (63). Individuals whose moral beliefs align with their C-P use may experience less psychological conflict or distress, decreasing the likelihood of perceiving their behaviour as problematic. Indeed, cultural context in which sexuality is expressively diverse could attenuate the association between SP and perceived PPU. Thus, the relationship in this study between PPU and SP may reflect both the normalisation of C-P consumption and the congruence between developmental normalisation, personal values and sexual attitudes.

Although gender did not moderate the association between PPU and SP, it emerged as a strong predictor of PPU, with men reporting substantially higher scores than women. Wright and Vangeel (11) found that men tend to be more sexually permissive than women, a pattern often interpreted through evolutionary psychology frameworks in which differential reproductive strategies shape sexual attitudes. Men may view uncommitted or short-term sexual encounters more favourably due to lower reproductive costs. However, descriptive analyses in the present study showed that women reported slightly higher SP than men. This may reflect shifting sexual norms and the influence of sex-positive feminist movements (64). Accepting attitude toward C-P has been associated with increased C-P consumption (65) and normative (sexual and social) pressures may shape women’s engagement with C-P. For some women, C-P may serve as a source for sexual education (62) or a non-judgmental space to explore sexual knowledge (61). Recent work suggests that young women often feel regulated in their sexual lives and may fear being perceived as “bad” or “defective” when deviating from expected norms (66, p. 1410), potentially contributing to increased C-P use. Li et al. (67) further found that sexual communication, rather than frequency of C-P use, mediated the relationship between positive attitudes toward C-P and sexual well-being among young women in the UK. These findings emphasise the importance of distinguishing between FC-PE, positive attitudes toward C-P and low SP. Gender-specific patterns in SP further highlight the need to explore how sexual attitudes differentially relate to PPU across genders. Although PPU varied across sexual orientation groups with participants identifying as homosexual reporting higher PPU, sexual orientation did not moderate the relationship between SP and PPU. The absence of a relationship may reflect insufficient statistical power due to uneven group sizes, or it may indicate the presence of resilience factors or value-congruent sexual scripts that prevent C-P use from becoming or felt as problematic (56). Future research with larger and more diverse samples is needed to clarify these patterns.

Several limitations should be considered when interpreting the findings. Although gender distribution was relatively balanced, the uneven representation of sexual orientation groups may have influenced results and limited generalisability. In the future, additional options for sexual orientation should be considered for more inclusive research outcomes. Cultural factors also constrain generalisability, as C-P laws, norms and attitudes vary widely across countries. The online survey format may have posed challenges for older participants with lower digital literacy, potentially affecting participation and data completeness. Future studies may benefit from mixed-method data collection (e.g., paper-based surveys, qualitative interviews) to improve accessibility. Self-report measures introduce additional limitations, including memory bias, especially for older participants recalling early exposure and social desirability bias given the sensitive nature of the topic. Finally, although this study used a validated measure of PPU, self-reported scores do not necessarily constitute a diagnosis of CSBD, as such diagnosis can only be made by a qualified clinician. It is important to specify that in the International Classification for Diseases (ICD-11), PPU is generally regarded as a manifestation of CSBD within the category of Impulse Control Disorders (68). As of 2025, the Diagnostic Statistical Manual of Mental Disorders (DSM-5-TR) does not recognise C-P addiction as a formal diagnosis. Hence, related distress may be conceptualised using alternative frameworks (e.g., compulsive or addictive behaviours, 69). Future research may consider incorporating additional measures of impulsivity or addictive tendencies for future assessments of PPU. Future research should also consider types of C-P content as potential predictors of PPU, by providing a more extensive list of response options to participants than those presented in this study.

Despite these limitations, the findings reinforce the importance of awareness and monitoring of C-P consumption. Frequent exposure may serve as an indicator of PPU, highlighting the value of early intervention to prevent negative outcomes for individuals and their social environments. In clinical or therapeutic settings, assessing FC-PE, loss of control and related behaviours may help identify individuals at risk and facilitate timely support. Interventions that reduce stigma and shame surrounding pornography-related difficulties may also encourage help-seeking. Consistent with this, a recent Independent Pornography Review recommends that the UK Commission conduct research and case studies to “reduce stigma around reaching out for help” (7, p. 27). The review was positively received by the UK Government, demonstrating proactive action to reduce the risks and harms associated with C-P in an effort to protect both children and adults (see 70). Self-monitoring strategies may assist individuals in managing consumption patterns and associated thoughts. Given the decreasing age of first exposure to C-P, these strategies should extend to educational settings where young people can receive guidance on online safety, responsible Internet use and managing exposure to sexually explicit media.

In conclusion, the current study examined the relationships between PPU, FC-PE, SP and the moderating roles of gender and sexual orientation among adult C-P consumers who reported first exposure before the age of 18. FC-PE emerged as a meaningful indicator of risk for developing PPU, particularly among men. These findings support the implementation of early preventative education focused on self-regulation and healthy coping strategies. The potential associations between PPU with either SP or sexual orientation highlight the need for further research exploring how sexual attitudes and identities shape pornography-related behaviours. While recent legal regulations aim to restrict minors’ access to C-P, continued research is essential to understand the mechanisms underlying PPU and to promote digital safety and well-being for all users.

## Data Availability

The raw data supporting the conclusions of this article will be made available by the authors, without undue reservation.

## Footnotes

1 LGBTQ stands for lesbian, gay, bisexual, transgender and queer communities.

## Appendix: Psychometric instruments

Problematic Pornography Consumption Scale (PPCS; 9)

Answer the following questions, rating them by how much you feel they apply to you (1 = Never, 2 = Rarely, 3 = Occasionally, 4 = Sometimes, 5 = Often, 6 = Very often, 7 = All the time).

1. I felt that porn is an important part of my life.
2. I used porn to restore the tranquility of my feelings.
3. I felt porn caused problems in my sexual life.
4. I felt that I had to watch more and more porn for satisfaction.
5. I unsuccessfully tried to reduce the amount of porn I watch.
6. I became stressed when something prevented me from watching porn.
7. I thought about how good it would be to watch porn.
8. Watching porn got rid of my negative feelings.
9. Watching porn prevented me from bringing out the best in me.
10. I felt that I needed more and more porn in order to satisfy my needs.
11. When I vowed not to watch porn anymore, I could only do it for a short period of time.
12. I became agitated when I was unable to watch porn.
13. I continually planned when to watch porn.
14. I released my tension by watching porn.
15. I neglected other leisure activities as a result of watching porn.
16. I gradually watched more “extreme” porn, because the porn I watched before was less satisfying.
17. I resisted watching porn for only a little while before I relapsed.
18. I missed porn greatly when I didn’t watch it for a while.

**Brief Sexual Attitudes Scale (BSAS;** 27**). Sexual permissiveness is measured from questions 1 to 10.**

For each statement select the response on the answer scale that indicates how much you agree or disagree with that statement (1 = Strongly disagree, 2 = Moderately disagree, 3 = Neutral, 4 = Moderately agree, 5 = Strongly agree). Some of the items refer to a specific sexual relationship, while others refer to general attitudes and beliefs about sex. Whenever possible, answer the questions with your current partner in mind. If you are not currently dating anyone, answer the questions with your most recent partner in mind. If you have never had a sexual relationship, answer in terms of what you think your responses would most likely be.

1. I do not need to be committed to a person to have sex with him/her.
2. Casual sex is acceptable.
3. I would like to have sex with many partners.
4. One-night stands are sometimes very enjoyable.
5. It is okay to have ongoing sexual relationships with more than one person at a time.
6. Sex as a simple exchange of favors is okay if both people agree to it.
7. The best sex is with no strings attached.
8. Life would have fewer problems if people could have sex more freely.
9. It is possible to enjoy sex with a person and not like that person very much.
10. It is okay for sex to be just good physical release.
11. Birth control is part of responsible sexuality.
12. A woman should share responsibility for birth control.
13. A man should share responsibility for birth control.
14. Sex is the closest form of communication between two people.
15. A sexual encounter between two people deeply in love is the ultimate human interaction.
16. At its best, sex seems to be the merging of two souls.
17. Sex is a very important part of life.
18. Sex is usually an intensive, almost overwhelming experience.
19. Sex is best when you let yourself go and focus on your own pleasure.
20. Sex is primarily the taking of pleasure from another person.
21. The main purpose of sex is to enjoy oneself.
22. Sex is primarily physical.
23. Sex is primarily a bodily function, like eating.
